# Volatile Transference and Antimicrobial Activity of Cheeses Made with Ewes’ Milk Fortified with Essential Oils

**DOI:** 10.3390/foods9010035

**Published:** 2020-01-01

**Authors:** Carmen C. Licon, Armando Moro, Celia M. Librán, Ana M. Molina, Amaya Zalacain, M. Isabel Berruga, Manuel Carmona

**Affiliations:** 1 Department of Food Science and Nutrition, California State University, Fresno, 5300 N Campus Drive M/S FF17, Fresno, CA 93740, USA; cliconcano@mail.fresnostate.edu; 2 Facultad de Ingeniería Agronómica, Universidad Técnica de Manabí, Avda. José María Urbina y Che Guevara, 130105 Portoviejo, Manabí, Ecuador; 3 Food Product Quality Department, Consum S. Coop, Av. Alginet s/n, 46460 Silla, Valencia, Spain; cmlibran@consum.es; 4 Food Quality Research Group, Institute for Regional Development (IDR), Universidad de Castilla-La Mancha, Campus Universitario, 02071 Albacete, Spain; ana.molina@uclm.es (A.M.M.); mariaisabel.berruga@uclm.es (M.I.B.); 5 Cátedra de Química Agrícola, E.T.S.I.A., Universidad de Castilla-La Mancha, Campus Universitario, 02071 Albacete, Spain; Amaya.zalacain@uclm.es; 6 School of Architecture, Engineering and Design, Food Technology Lab, Universidad Europea de Madrid, C/Tajo s/n, Villaviciosa de Odón, 28670 Madrid, Spain

**Keywords:** cheese, essential oils, *Escherichia coli*, *Clostridium tyrobutyricum*, *Penicillium verrucosum*, antimicrobial

## Abstract

During the last decades, essential oils (EOs) have been proven to be a natural alternative to additives or pasteurization for the prevention of microbial spoilage in several food matrices. In this work, we tested the antimicrobial activity of EOs from *Melissa officinalis*, *Ocimum basilicum*, and *Thymus vulgaris* against three different microorganisms: *Escherichia coli*, *Clostridium tyrobutyricum*, and *Penicillium verrucosum*. Pressed ewes’ cheese made from milk fortified with EOs (250 mg/kg) was used as a model. The carryover effect of each oil was studied by analyzing the volatile fraction of dairy samples along the cheese-making process using headspace stir bar sorptive extraction coupled to gas chromatography/mass spectrometry. Results showed that the EOs contained in *T. vulgaris* effectively reduced the counts of *C. tyrobutyricum* and inhibited completely the growth of *P. verrucosum* without affecting the natural flora present in the cheese. By contrast, the inhibitory effect of *M. officinalis* against lactic acid bacteria starter cultures rendered this oil unsuitable for this matrix.

## 1. Introduction

The cheese microbiota has an important role in the development of cheese flavor and texture. By contrast, exogenous microorganisms can have a negative impact on the organoleptic properties of cheese, with the potential for great economic loss. For example, the occurrence of coliforms (*Escherichia coli*, *Klebsiella aerogenes*) and sporulating butyric bacteria (*Clostridium tyrobutyricum*, *C. butyricum*, and *C. sporogenes*) is known to be responsible for early and late cheese blowing, respectively [1,2]. Also, some filamentous molds (*Penicillium comune*, *P. verrucosum*, and *P. nalgiovense*) of the dairy factory environment [3,4], which are usually found in cheese rind or interior, have been associated with the presence of mycotoxins, with a consequent human health risk [5,6]. Late cheese blowing is quite frequent in semi-hard and hard cheeses, including Grana Padano, Cheddar, and Manchego [7,8,9,10], and is characterized by the presence of numerous and irregular internal holes produced by CO_2_ released from lactate metabolism [7,11]. In this context, *C. tyrobutyricum* is considered as a main spoiler agent markedly affecting the volatile profiles of cheese [12].

Several approaches are available to reduce the occurrence of late blowing cheese spoilage, such as pasteurization or the use of additives, including nitrates and lysozyme; however, none of these approaches is ideal. In the case of pasteurization, bacterial endospores can survive the pasteurization process and germinate as vegetative cells in cheese during ripening. Also, the addition of nitrates has been associated with the presence of nitrosamine in cheese, although the European Food Safety Authority has recently re-assessed the acceptable safe daily intake of nitrites and nitrates [13]. Lastly, lysozyme has antimicrobial effects on lactic acid bacteria during cheese ripening [14]. Given these constraints, the use of essential oils (EOs) as natural food preservatives has steadily gained recognition as an alternative to the aforementioned treatments, as they are designated as “Generally Recognized as Safe” by the Food and Drug Administration [15,16], and they have proven antibacterial [17] and antifungal [18,19] activity. That being said, the antimicrobial activity of EOs has been assayed mostly under in vitro conditions and against pathogenic microorganisms [20,21], and there is a paucity of studies focusing on food products, especially in cheese [22,23]. In this context, Hyldgaard et al. [15] have emphasized the importance of understanding the behavior of EOs in a food matrix—as differences have been reported between plant and animal food products [24,25]. Moreover, there is conflicting evidence between studies, even when using the same product type, likely because of compositional differences, for example, cheeses with different fat or moisture content [5,23,26]. The utility of EOs or their compounds in cheese production has been examined in several studies [22,23], including their use as surface covers [5], or added directly to a finished product [19,21,26] or microencapsulated [27]. Yet, very little is known about the impact of adding EOs directly to milk before cheesemaking. Hamedi et al. [28] showed that the efficacy of EOs against *Salmonella* spp. in cheese diminished significantly when the results were compared with those obtained using a laboratory medium. It would be reasonable to expect that the EOs used to combat spoilers or pathogens should also be tested against lactic acid bacteria and different starter cultures required for semi-hard and hard cheese making.

Against this background, the present study was designed to determine the antimicrobial activity and the transfer of chemical compounds to fortified cheeses of different EOs. We used *Melissa officinalis* (lemon balm), *Ocimum basilicum* (sweet basil), and *Thymus vulgaris* (common thyme), and three typical cheese spoilers, *E. coli*, *C. tyrobutyricum*, and *P. verrucosum*.

## 2. Materials and Methods

### 2.1. Plant Material and EO Production

The aerial parts of *M. officinalis*, *O. basilicum*, and *T. vulgaris* were supplied by Nutraceutical SRL (Brazov, Romania). The raw material was packed in sealed plastic bags and stored in the dark at room temperature until analysis. EOs were obtained by solvent-free microwave extraction (SFME) with a NEOS^®^ apparatus (Milestone, Sorisole, Italy) using methodology previously employed by Moro et al. [29]. In total, 150 g of the plant was placed in the NEOS reactor with 250 mL of Milli-Q water to wet the dry plant sample. As its name implies, the technique does not use a solvent, but the plant must contain the water that drags the essential oils when heated by microwaves, the principle with which this equipment works. Exhaustive extraction of EOs was then performed (35 min): the extraction power was set at 600 W (5 min) and then at 250 W (30 min), and the temperature was monitored with an infrared sensor for avoiding overheating (95 ± 5 °C). The oil was collected in the device with graduation marks available to the equipment itself for this purpose. For the antimicrobial activity test, the EOs were filtered using 0.2-μm PTFE syringe filters (Millipore, Madrid, Spain) to ensure the absence of microorganisms before use.

### 2.2. Milk Samples

“Manchega” breed ewes’ milk was used for cheese fabrication. Bulk tank milk was collected from a commercial farm in Albacete (Spain). Milk had the following compositional values (g/100 g): dry matter, 17.81; fat content, 6.80; and protein content, 5.61. The mean pH was 6.66, somatic cell counts were 603 × 10^3^ cells/mL and 158 × 10^3^ CFU/mL microbial load.

### 2.3. Microbial Strains

The following assayed strains were purchased from the Spanish Type Culture Collection (CECT, Burjassot, Valencia, Spain): *E. coli* CECT 4201, *C. tyrobutyricum* CECT 4011, and *P. verrucosum* CECT 2906.

### 2.4. Elaboration of Cheese Samples Fortified with EOs

Before beginning cheesemaking, vats of 30 L of milk were fortified with EO samples at a final concentration of 0.250 g/kg. EOs were mixed 1:1 with a commercial food emulsifier (Tween-20^®^ Food quality, Panreac, Spain) selected because it is considered safe [30]. The control vat contained the emulsifier at the same concentration used in the experimental (EOs) tanks. Milk was heated to 20 °C for 30 min to facilitate oil solubilization, and a pressed ewes’ milk cheese procedure was performed at a pilot dairy plant from Castilla-La Mancha University, according to Licón et al. [31], with some modifications. Briefly, the starter culture (CHOOZIT MA4001; Danisco, Sassenage, France) was added for 30 min with stirring, and the temperature was increased to 30 °C. At this point, commercial rennet (0.023% *v*/*v*) was added to the vat with vigorous stirring, and the milk was allowed to coagulate. Thirty minutes later, the curd was cut into 8–10 mm cubes, heated (37 °C), and stirred for 45 min before whey separation. Curd was press-molded for 4 h until reaching pH 5.2. Lastly, cheeses were salt brined at 9 °C and stored in a ripening chamber over four months at 12 °C and 80% humidity prior to performing the assays. The cheese chemical composition was determined using a Foss FoodScan analyzer (FoodScan Lab, FOSS, Hillerød, Denmark).

### 2.5. Volatile Extractions and HS-SBSE/GC/MS Analyses

EOs were directly injected (0.2 μL) into a gas chromatograph following the methodology of Moro et al. [29]. Milk and cheese volatile extraction was performed by the headspace stir bar sorptive extraction (HS-SBSE) method. For the former, 10 mL liquid dairy samples (milk and whey) were pipetted separately into headspace glass vials, whereas cheese volatile extraction was performed following the methodology of Licón et al. [32]. For all dairy samples, headspace glass vials were affixed with inserts for headspace exposition and supplemented with a 1 × 10^−3^ g/kg aqueous solution of the internal standard ethyl octanoate (Aldrich Chemical Co., Milwaukee, WI, USA). A polydimethylsiloxane (PDMS)-coated stir bar (0.5 mm film thickness, 10 mm length in liquid samples, and 20 mm length in cheese samples; Twister, Gersterl GmbH, Mülheim an der Ruhr, Germany) was placed into the insert, and headspace vials were sealed with an aluminum crimp cap. Before analysis, the glass inserts and vials were thoroughly cleaned and heat conditioned at 110 °C to avoid any odorous contamination. The extraction of volatile compounds was performed following conditions proposed by Moro et al. [33], stirring at 1000 rpm for 120 min (milk and whey) or 240 min (cheese) at 45 °C. The PDMS stir bars were rinsed with distilled water, dried with cellulose tissue, and finally transferred into thermal desorption tubes for the GC/MS analysis.

The extracted volatiles from dairy samples were desorbed in an automated thermal desorption system (Turbo Matrix ATM, PerkinElmer, Norwalk, CT, USA) under the following conditions: oven temperature, 280 °C; desorption time, 5 min; cold trap temperature, −30 °C; helium inlet flow rate, 45 mL/min. The volatiles were transferred into a Varian CP-3800 gas chromatograph (GC) equipped with a Saturn 2200 ion trap mass spectrometer (MS) (Varian Inc., Palo Alto, CA, USA) and an Elite-Volatiles Specialty phase capillary column (30 m × 0.25 mm i.d., 1.4 μm film thickness; PerkinElmer, Shelton, CT, USA). The column temperature was set at 35 °C for 2 min and then raised at 5 °C/min to 240 °C and held for 5 min. The detector temperature was 250 °C, and the helium carrier gas flow rate was 1 mL/min. The electron ionization mode at 70 eV was used for the MS analysis. The mass range varied from 35 to 300 *m*/*z*.

To avoid matrix interferences between the EOs and dairy matrix volatiles, the MS identification of volatiles was performed in single-ion-monitoring mode using their characteristic m/z values and by comparison of their mass spectra with those of pure compounds or reported in the NIST/ADAMS library. The identities of the EO components were established from the GC retention time (relative to Kovats index). Quantification was carried out in scan mode and expressed as the relative area using the correction factor for the internal standard (ethyl octanoate) area. The results of each volatile compound that was transferred to the dairy matrix were expressed as relative concentration area (g/kg) using the internal standard correction factor. Then the transference ratio or recovery yield (%) from milk to cheese of each compound that was found was calculated by the following Formula (1):

recovery yield (%) = [Xi (g/kg)/X (g/kg)] × 100 (1)

where Xi indicates the presence of each compound in cheese, and X indicates the presence of the same compound in milk. Dairy samples were analyzed in triplicate.

### 2.6. Cheese Microbial Content

To enumerate the microbial content on ripened cheeses, a 10-g sample of each cheese was aseptically homogenized with 90 mL of sterile 0.1% (*w*/*v*) peptone water in an IUL Stomacher (IUL SA, Barcelona, Spain) for 60 s. Serial decimal dilutions of the homogenates were prepared with buffered peptone water (BPW) (Scharlau, Barcelona, Spain) and plated onto the corresponding media in duplicate using an Eddy Jet spiral plater (Eddy Jet v1.23, IUL SA, Barcelona, Spain). Total aerobic bacterial counts were performed on plate count agar (PCA; Panreac Química S.L.U., Barcelona, Spain) after incubation at 32 °C for 48 h under aerobic conditions. Lactic streptococci were plated on M17 agar (Biokar Diagnostics, Barcelona, Spain) with incubation at 37 °C for 48 h, under aerobic conditions. Brilliant Green Bile Agar was used for coliform incubation (BGB; Pronadisa Conda, Madrid, Spain) at 37 °C for 24 h, under aerobic conditions. *Clostridium* spp. was plated on a reinforced clostridial agar (RCA; Oxoid, Basingstoke, UK) and incubated at 37 °C for 48 h, under anaerobic conditions. Molds and yeasts were seeded in potato dextrose agar (PDA; Merck, Darmstadt, Germany) and incubated at 25 °C, during 96 h, in aerobic conditions. Microbial growth estimations were done with an automatic plate counter (Countermat Flash 4.2, IUL Intruments S.A., Barcelona, Spain), and the results were expressed as log cfu/g.

### 2.7. Antimicrobial Activity Test

**Figure 1:**
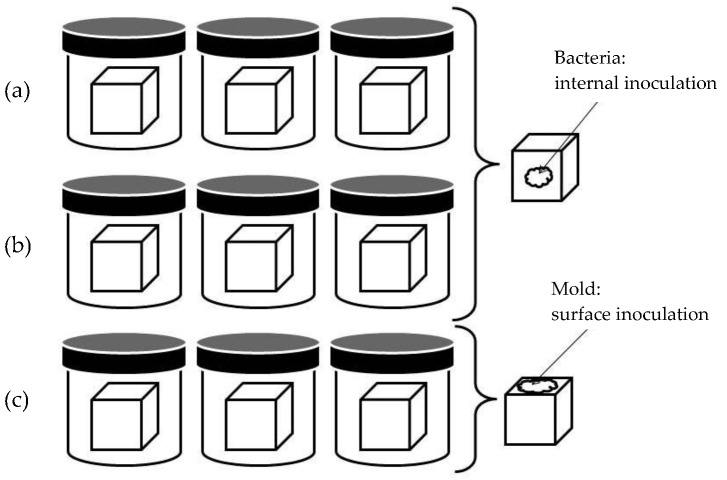
Antimicrobial activity assay performed by the inoculation of fortified cheeses with: (**a**) *Clostridium tyrobutyricum* (37 °C, anaerobic conditions), (**b**) *Escherichia coli* (37 °C, aerobic conditions) and (**c**) *Penicillium verrucosum* (25 °C, aerobic conditions).

The experimental procedure for antimicrobial activity determination is depicted in Figure 1, and allows the investigation of microbial spoilage, in the case of an external contamination such as that occurring in ripening chambers with molds. Nine cheese cubes of 27 mm^3^ were obtained from each cheese using a cheese blocker (BOSKA, Bodegraven, Holland). The cubes were divided into three subgroups, with three cubes in each. Cubes were introduced into a sterile container and distributed as follows: Group 1, internal inoculation with *C. tyrobutyricum* at 10^3^ cfu/g, incubated at 37 °C under anaerobic conditions (AnaeroGenTM, Oxoid LTD., Basingstoke, UK); Group 2, internal inoculation with *E. coli* at 10^3^ cfu/g, incubated at 37 °C under aerobic conditions; Group 3, surface inoculation with *P. verrucosum* at 10^3^ cfu/cm^2^, incubated at 25 °C under aerobic conditions. *P. verrucosum* was inoculated onto the surface, given its inability to grow in the interior of the cheese.

### 2.8. Microorganism Inoculum Preparation

*C. tyrobutyricum* spore suspensions were obtained by prior prolonged incubation (1 week) on Reinforced Clostridial Medium (Oxoid LTD.). Subsequently, spores were harvested and cleaned following a procedure adapted from Yang et al. [34], which briefly consisted of double purification by centrifugation at 8000× *g* for 15 min at 4 °C. The final pellet was resuspended in sterilized distilled water, and the spore concentration of the suspension was determined by adapting the procedure of Anastasiou et al. [35] after 15 min heat treatment at 80 °C, by serial dilution in BPW. An *E. coli* suspension was obtained after 22 h of cultivation on Triptone Soy Medium (Oxoid LTD.); the colony-forming units were also established by serial dilution in BPW. In both cases, 1 mL aliquots of concentrated bacterial suspensions were stored at −20 °C in 15% of glycerol until needed for inoculation at a final concentration of 10^3^ cfu/g.

*P. verrucosum* spore suspensions were sub-cultured weekly on Potato Dextrose Agar (Merck, Darmstadt, Germany) at 25 °C in the dark. Conidia were harvested according to Baratta et al. [36], and the spore suspension was adjusted to an optical density of 0.5 (λ = 530 nm), equivalent to 10^5^ spores/mL. This suspension was employed for the immediate surface inoculation of cheese samples at a concentration of 10^3^ cfu/g.

After 1 week of incubation, starters, total viable counts, and target microbial growth were determined in all cheese cubes. The experiment was performed in duplicate.

### 2.9. Statistical Analysis

Descriptive analysis and analysis of variance (ANOVA; *p* < 0.001) coupled to a Tukey’ test (*p* < 0.05) were performed to determine group differences between the antimicrobial activity results using IBM Statistics SPSS software, v24 (SPSS Inc., Chicago, IL, USA).

## 3. Results and Discussion

### 3.1. Extraction and Composition Analysis of Essential Oils

EOs from aromatic plants are a complex mixture of volatile oils of low molecular weight that are obtained by steam distillation [37]. In the present study, EOs were obtained using a modern extraction technique based on solvent-free, microwave hydrodiffusion, also known as SFME or microwave hydrodiffusion and gravity [38]. The use of this technique offers several advantages over conventional hydrodistillation or solvent distillation, including the avoidance of artefacts during distillation, and also savings in energy and extraction time [38].

Chemical characterization of the EOs in terms of volatile composition was necessary before determining the transference ratio during cheesemaking. The total number of compounds identified in the EOs ranged from 14 in *O. basilicum* to 27 in *T. vulgaris* (Table 1), and they constituted over 87% of the total area composition.

According to chemical families of compounds, all EOs were represented mainly by monoterpenes—with 83.80% to 96.57% of the total peak area, respectively. Sesquiterpenes represented <3.2% of the total composition. In accordance with our previous study [33], the present results showed that all of the EOs were dominated by two or three major compounds (Table 1), representing up to 40% of the total area. These main compounds were commonly oxygenated monoterpenes, terpenes, which undergo biochemical modifications that add oxygen molecules and move or remove methyl groups [15]. In contrast to other studies [17,18], we found that the *O. basilicum* EO was described mainly by the aromatic compound 4-allyl-anisole also known as methyl chavicol (58.21%), rather than linalool (11.21%), which has been reported in larger amounts by other authors (20%–66%). In addition, we found a small amount (3.20%) of the sesquiterpene α-bergamotene (E)(Z).

Linalool is a linear monoterpene that is frequently found in volatile plant extracts. We found this in a range from 1.71% to 34.54% of the total area; the latter case was found for *T. vulgaris*, exceeding the concentration of thymol, which is usually the characteristic EO marker of this species [20,39]. The other family groups of compounds identified in this EO represented ~2% of the total composition.

Regarding the EOs of *M. officinalis*, nerol (35.85%) and neral (35.34%) were the major compounds identified, and the remaining compounds did not exceed 2.7% of the total area. These results differ from those of previous works [40,41], which suggested that citral—a mixture of neral and geranial—is the major compound [40,41]. Geranial and nerol are biosynthetically connected, as geranial is the aldehyde isomer of nerol.

The absence of or a smaller-than-expected amount of compounds has been reported by other authors, such as the absence of thymol in thyme oil, and the presence of other compounds, such as carvacrol, a phenolic monoterpene, or p-cymene, and γ-terpinene, precursors in its biogenetic pathway [42]. In this regard, some authors have highlighted the effect of plant chemotype on EO composition for the presence of thymol, thymol/linalool, and carvacrol chemotypes in different varieties of thyme [43]. Moreover, several studies have emphasized the importance of culture-growing conditions and harvesting, in addition to different varieties, when EOs are chemically characterized [17,42]. The extraction methodology is also known to affect the composition and quality of extracts, as the use of high temperatures can stimulate the hydrolysis and polymerization of some esters [44], whereas the use of solvents can leave residual substances that affect the biological properties of EOs [45]. Using the same extraction procedure as that used here, Okoh et al. [46] achieved better extraction yields and larger amounts of oxygenated monoterpenes than with EOs obtained by hydrodistillation, which may explain the compositional differences between studies.

### 3.2. Volatile Composition of Dairy Samples

As previously reported by Tajkarimi et al. [47], the normal concentration range for spices and herbs used in food systems is between 0.05% and 0.1%. In the present study, an EO concentration of 0.25 g/kg was chosen to study the transference of volatile compounds during the cheese-making process, to prevent an excessive sensory impact and to provide antimicrobial activity. Indeed, the concentration of EOs is an important consideration, as it has been demonstrated that they may have an undesirable impact on cheese sensory properties by modifying the dynamics or activity of the microbial ecosystem during cheese making and ripening. This hypothesis derives from indirect observations in several trials of hard-cooked cheeses and experiments performed by Tornambé et al. [48], where EO concentration levels higher than 10 g/kg resulted in a high sensory impact and consequent rejection by consumers. Because specific surfactant actions are required to improve the affinity of the matrix for volatile compounds, particularly terpenes, we selected Tween^®^-20 as a polysorbate surfactant, whose stability and relative lack of toxicity allow it to be used as a detergent and emulsifier for culinary, scientific, and pharmacological purposes.

The methodology selected for the extraction and characterization of volatiles (HS-SBSE coupled with GC/MS) is a common technique in food volatile analysis, and it has been specifically optimized by Licón et al. [32] and Moro et al. [33] for pressed ewes’ milk cheeses. This food matrix is quite complex, and several interactions can potentially take place between food components and EOs [15] due to the high fat and protein content of the cheese. For the present study, we only examined the volatiles present in the EOs, and the identification of other cheese compounds was dismissed. The results of the concentration of the main compounds identified in milk, cheese, and whey, together with the carryover percentages, are provided in Table 2.

The major compounds of the EOs (Table 1) corresponded to those identified in larger quantities in milk, cheese, and whey, whereas the minor compounds were below the method’s limit of detection. The number of detected compounds in the different matrices ranged from 9 to 22, and between 82% and 95% of the compounds detected in the EOs were transferred to the dairy products. This transfer range was much broader than that described by Tornambé et al. [48] (43%) when a pasture plant EO was added to milk.

Regarding the different chemical families found in milk, monoterpenes were the most abundant in milk spiked with *M. officinalis* (47.76 mg/kg), *T. vulgaris* (249.81 mg/kg), and *O. basilicum* (82.71 mg/kg). For cheese and whey, different transference rates were obtained for each plant: for *M. officinalis*, monoterpene compounds (7.06%) in cheese and sesquiterpenes (30.61%) in whey showed the lowest and the highest carryover effects in this plant; for *T. vulgaris*, sesquiterpenes (16.67% and 39.58%) were the most abundant family of compounds in cheese and whey, respectively; whereas for *O. basilicum*, the best carryovers were observed for monoterpenes (28.44% and 23.15%) for cheese and whey, respectively. Transference of compounds in EOs to dairy matrices is challenging, as they are known to interact with fat, carbohydrate, and protein matrices in cheese [20,24]. Specifically, proteins and whey proteins can interact with compounds presenting with a hydroxyl group, restricting their ability to be transferred [20,23].

As individual compounds, the major content of *M. officinalis*-enriched dairy products (milk, cheese, whey) were nerol (17.56, 0.86, 3.57 mg/kg), neral (16.30, 0.86, 3.38 mg/kg), and camphene (8.60, 0.99, 1.36 mg/kg). Most of the compounds identified in *O. basilicum*-enriched milk were below 0.60 mg/kg, with the exception of 4-allyl-anisole (47.02 mg/kg), 1,8 cineole (15.49 mg/kg), and linalool (13.99 mg/kg). For *T. vulgaris*-enriched dairy products, a larger abundance of significant compounds was found, as eight compounds >10 mg/kg were detected in milk, reaching 6.5 mg/kg in cheese, and as high as 13 mg/kg in whey. The same was found for cheese and whey. However, these individual major compounds did not offer the best carryover ratios, and other minor compounds were better transferred: linalool (14.29%) in cheese, and β-caryophyllene (30.61%) in whey from *M. officinalis*, β-caryophyllene (16.67%) in cheese and 1,8 cineole (47.12%) in whey from *T. vulgaris*, and α-thujene (75.00%) in cheese and γ-terpinene (30.00%) in whey from *O. basilicum*. In the case of α-thujene, it has to be pointed out that it is a high transfer rate but for a very minority compound, which we do not even find in the essential oil of this plant. Maybe the enzymatic activity present in the milk could convert sabinene into α-thujene since they have great structural similarity. Indeed, it seems that the different functional groups of compounds also affected the transfer ratios, which were better for hydrocarbon monoterpenes than for oxygenated ones. Thus, better carryover ratios were reached by using EOs that are richer in hydrocarbons rather than oxygenated monoterpenes.

### 3.3. Antimicrobial Activity

The established concentration mean value of 10^3^ was decided as a mid-point of known studies for the different species. In the case of *P. verrucosum*, the studies considered were those of Nielsen et al. [49] and Vazquez et al. [5]. The first ones inoculated Arzua-Ulloa cheeses with fungal species at the concentration of 1.5 × 10^3^ spores/cm^2^ and the second ones at 10^2^ spores/cm^2^. We decided to fit the inoculum at an intermediate level of 10^3^ cfu/cm^2^. For *E. coli*, several authors [21,50,51] used contamination levels in cheese or milk for cheese elaboration in the range from 10^1^ cfu/g or mL to 10^5^ cfu/g or mL. The average value of 10^3^ seemed reasonable again, as it was also somewhat below the maximum contamination levels found for Clostridium in cheeses by several authors [9,52].

**Figure 2:**
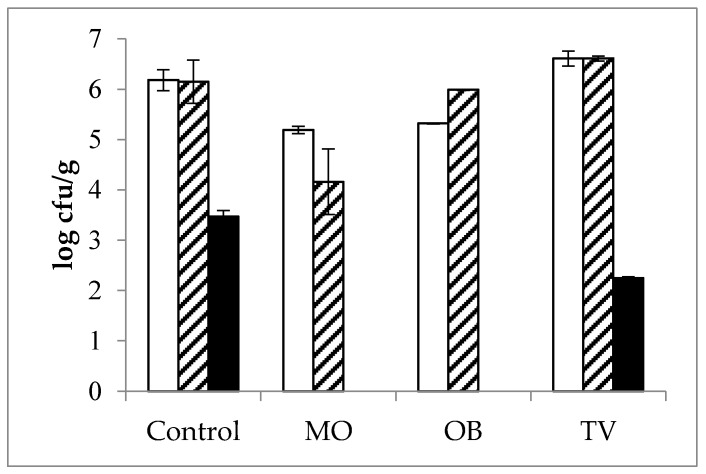
Microbial content (log cfu/g; mean ± SEM) in the control, *Melissa officinalis* (MO), *Ocimum basilicum* (OB), and *Thymus vulgaris* (TV) ripened cheeses. (Total Viable Counts: 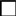; Lactic Acid Bacteria: 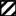; *Clostridium* spp.: 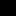).

The antimicrobial effects of the plant EOs on the initial flora of fortified cheeses are shown in Figure 2. The antimicrobial effect of *M. officinalis* EOs was strong, whereas the effect of *T. vulgaris* EOs was milder, and the effect of *O. basilicum* EOs was intermediate. Additionally, *M. officinalis* and *O. basilicum* EOs showed the greatest inhibitory effect against clostridia microorganisms naturally occurring in the milk and cheese. Specifically, the EOs from *M. officinalis* and *O. basilicum* completely blocked the growth of Clostridium spp., whereas *T. vulgaris* tempered the growth of these bacteria by more than 1 log unit (2.25 and 3.47 log cfu/g in the *T. vulgaris*-fortified and control cheese, respectively). However, it was not possible to evaluate the inhibitory capacity on initial coliforms or molds as the milk was free of these two groups of microorganisms since none of them grew even in control cheeses.

These findings indicate that late cheese blowing caused by clostridia development can be prevented by the tested EOs. Nevertheless, the robust antibacterial effect of *M. officinalis* EOs might negatively affect cheese ripening as it greatly influenced normal cheese flora development by reducing the starter bacteria content by nearly 2 log units (Figure 2). This imbalance in lactic streptococci might lead to flat flavors due to their lower activity in the ripening stages [53], paste defects deriving from slow acidification during cheese preparation [54], or even early cheese blowing as lactose consumption competition with coliforms would be lacking [55]. Indeed, when producing cheese, delays of more than 30 min were observed during the *M. officinalis* acidification process (data not shown). As mentioned, it was impossible to ascertain the effect of these EOs on coliforms, probably owing to the water activity of the four-month ripened cheeses preventing bacterial growth. Moreover, when compared against the control and *T. vulgaris*-fortified cheese, which had normal counts in a 150-day ripened cheese [56], the *O. basilicum* EOs had a mild effect on normal cheese flora (Figure 2).

**Figure 3:**
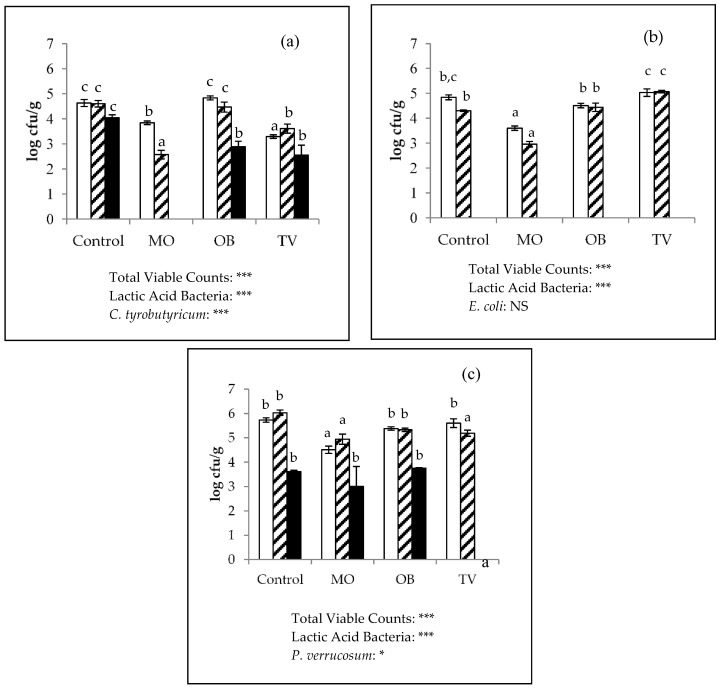
Microbial content (log cfu/g; mean ± SEM) in the control, *Melissa officinalis* (MO), *Ocimum basilicum* (OB), and *Thymus vulgaris* (TV) ripened cheeses inoculated and incubated for 1 week with (**a**) *Clostridium tyrobutyricum*, (**b**) *Escherichia coli*, and (**c**) *Penicillium verrucosum*. (Total Viable Counts: 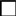; Lactic Acid Bacteria: 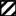; Target microorganism: 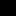). ***, *, NS: Significance level *p* < 0.001, *p* < 0.05 and non-significant, respectively. a, b, c: Different values among the same microbial group are significantly different between essential oils applications (*p* < 0.05).

The antimicrobial activity results of the fortified and control cheese samples after one week of incubation are shown in Figure 3. The effect of EOs on *Clostridium* spp. remained relevant (Figure 3a). In the Group 1 cubes (inoculated with *C. tyrobutyricum*), the addition of *O. basilicum* and *T. vulgaris* reduced the clostridial counts by more than 1 log unit as compared with the control samples (4.04 log cfu/g), whereas the *M. officinalis* cheeses had no clostridial counts. In our previous study on the anticlostridial activity of *M. officinalis* EOs in laboratory media, we found that the concentration of these EOs required to achieve total inhibition was ten times lower [57]. These results are in accordance with the fact that higher concentrations of EOs are needed in food matrices compared with those used in in vitro testing, highlighting the importance of performing simultaneous studies in vitro and in situ [58]. This inhibitory effect on clostridial growth reached in this assay was more robust than that described by other authors such as Deans and Ritchie [59], who tested pure oils in vitro, and were unable to demonstrate inhibition of C. sporogenes with any of the three tested EOs. By contrast, Baratta et al. [36] reported inhibitions with *O. basilicum* oil on another clostridial species, *C. perfringes*, which overall suggests varying resistance among strains.

No growth was recorded for any of the cubes in Group 2 (inoculated with E. coli), which fits with the initial cheese enumeration of the coliforms (Figure 2 and Figure 3b). It is commonly accepted that Gram-negative bacteria are more resistant than Gram-positive bacteria to EOs [23]. However, the results herein do not match with these observations, likely due to the harsh conditions of matured cheeses until coliform development; for instance, low pH, water activity, or lactose exhaustion [54].

Regarding the antifungal effect against *P. verrucosum*, we found a complete inhibition of growth in the *T. vulgaris*-fortified cheese, a slight reduction in the *M. officinalis* cheese (0.61 log unit) and no effect in the *O. basilicum* cheese (Figure 3c). These findings contrast with those obtained under in vitro conditions, where *O. basilicum* activity was the greatest, and *T. vulgaris* activity was the lowest [57]. Thus, the comparison of the effects of EOs on a cheese matrix and on laboratory media is important, as the activity may completely change.

Indeed, the activity of these EOs followed the same pattern in the cheese matrix as that observed in culture media against *C. tyrobutyricum*; thus, M. officinalis proved the most active, followed by *O. basilicum* and then *T. vulgaris* [60]. Cheese type can also have an effect on the antimicrobial potential of EOs, which was highlighted by Vázquez et al. [5], who found different effects of EO compounds when applied as cheese covers depending on cheese type. The authors of this study observed that it is possible to robustly inhibit *P. citrinum* in Arzúa-Olloa cheese with 200 μL/mL of eugenol, whereas no inhibition was observed for Cebreiro cheese, and the same was found when using thymol, the principal constituent of thyme oil [23,61]. These authors had to apply pure thyme oil to inhibit *Aspergillus parasiticus* growth in culture media.

Some other factors relating to the cheese matrix can completely alter the activity of EOs, which are in the main reduced as compared with laboratory media [24]. Several studies have demonstrated that food composition has a negative impact on EO efficacy, particularly carbohydrate, protein, and fat content [23,58]. In this line, low-fat cheeses are better for the action of EOs against Gram-positive bacteria but are worse for Gram-negative ones [26], and carbohydrates reduce the activity of EOs in other food matrices [24].

With the exception of the cheese samples incubated at 25 °C under aerobic conditions, the total viable counts and lactic streptococci generally decreased in relation to the initial cheese content (Figure 2 and Figure 3). The decline in these bacterial counts ranged from 0.1 to 3.3 log units. Furthermore, these reductions seemed to be influenced by not only the addition of EOs but also by the incubation conditions (Figure 3). Indeed, the combined effect of an anaerobic environment and the addition of *M. officinalis* or *T. vulgaris* EOs led to the most marked reductions in microbial flora (Figure 3a). During a long ripening period, like that studied in this work, a reduction in starter microorganisms is due not only to their loss of viability but also to the release of intracellular enzymes [62]. These starter microorganisms, which are stored refrigerated for a long ripening period, generally acclimatize to low temperature. Hence, this selection for more cold-tolerant microorganisms can explain the lower inhibition noted in the cheese cubes incubated at lower temperatures. In addition, increasing the incubation temperature from 25 °C to 37 °C can trigger the evaporation of the volatile compounds transferred from EOs to cheese, thus increasing their content in the vapor phase and, consequently, inhibiting bacteria more efficiently, as formerly observed by other authors [63,64].

## 4. Conclusions

The present study demonstrates that most of the compounds present in the EOs from *M. officinalis*, *T. vulgaris*, and *O. basilicum* were transferred from milk to cheese and whey. The carryover results show hydrocarbon monoterpenes to be the best transferred compounds from milk to cheese (11%–53%) and whey (11%–20%), indicating that they are less affected by fat and casein matrices. Obtaining dairy products supplemented with aromatic compounds enhances their flavor, but also contributes to bioactive properties (antioxidant or antimicrobial) and are alternatives for the dairy industry. Therefore, further research is recommended to test these potential properties. This work also demonstrates the importance of conducting specific studies on the target food matrix in order to evaluate the antimicrobial activity of EOs. Occasionally their efficacy could be extrapolated, which was the case of the three EOs studied against *C. tyrobutyricum*, although lower concentrations are required when assaying in culture media. Yet with other microorganisms like *P. verrucosum*, extrapolation can lead to a misinterpretation of the potential of these EOs if only in vitro assays are performed to select the most appropriate ones because many matrix factors can impact the results.

The effect of these EOs on microorganisms that are crucial for proper cheese ripening must also be considered, given the risk of converting a good, natural solution for a technological problem into a new limitation. By considering these considerations, and the concentrations assayed, we conclude that the EOs of *M. officinalis* and *O. basilicum* display excellent activity that helps combat microorganisms that may cause late cheese blowing before and after inoculation, and they do not show post-inoculation inhibition against mold. However, the *M. officinalis* EOs are not recommended because they potently inhibit the starter cultures usually added during cheese manufacture. The most balanced EOs for combating the microbial cheese defects addressed in this work are those of *T. vulgaris*, which reduce the clostridia content, strongly inhibit mold growth, and do not damage lactic streptococci starters. Further studies are needed to better understand the precise effect of EOs from aromatic plants on cheese matrices to adjust the most adequate EOs concentration for consumer acceptability, as well as their effect on different cheese varieties or ripening stages.

## Figures and Tables

**Table 1:** Volatile composition of essential oils (EOs) expressed as relative area (%).

Compounds	RT (min)	KI exp. *	*m*/*z* Pattern **	*Melissa officinalis*	*Ocimum basilicum*	*Thymus vulgaris*
**Number of compounds**				18	14	27
**Monoterpenes family**						
α-thujene	28.34	924	77/**93**/136	-	-	0.43
α-pinene	28.86	937	**93**/136	0.51	0.27	4.74
camphene	29.56	946	**93**/121/136	2.71	-	1.69
sabinene	30.51	970	**93**/77/41	0.02	0.23	1.27
β-pinene	30.77	975	41/**93**/107/121	2.22	0.52	8.13
myrcene	30.83	988	**41**/93/69	-	0.31	-
α-phelandrene	31.74	1002	**93**/77/136	-	-	0.8
α-terpinene	32.22	1014	**93**/121/136	-	-	5.12
β-ocimene (Z)	32.55	1017	79/**93**/136	0.43	-	-
p-cymene	32.64	1020	91/**119**	-	-	6.96
sylvestrene	32.66	1024	**41**/68/93/136	0.48	-	-
1,8 cineole	32.80	1026	43/108/139/**154**	-	5.85	-
β-phelandrene	32.90	1025	77/**93**/136	-	-	1.52
β-ocimene (E)	33.06	1032	79/**93**/136	0.31	0.84	0.11
γ-terpinene	33.74	1054	77/93/121/**136**	-	-	7.5
4-thujanol (Z)	34.16	1065	43/71/93/**139**/154	-	-	1.44
terpinolene	34.83	1086	43/**93**/121/136	0.3	-	2.47
linalool	35.09	1089	43/**71**/154	1.71	11.21	34.54
perillene	35.14	1093	**41**/69/81/150	0.12	-	-
4-thujanol (E)	35.37	1098	43/71/93/**139**/154	-	-	0.18
citronellal	37.04	1148	**41**/69/95/121/154	0.65	-	-
camphor	37.64	1141	**41**/95/152	-	0.54	0.3
borneol	38.01	1165	**95**/154	-	-	2.77
terpinen-4-ol	38.30	1174	43/**71**/154	0.28	0.33	10.54
α-terpineol	38.75	1186	43/**59**/93/136	0.56	-	2.14
4-allyl-anisole	38.84	1189	77/121/**148**	-	58.21	-
dihydro carvone (E)	39.09	1194	41/**67**/95/152	-	-	0.51
dihydro carvone (Z)	39.41	1200	41/**67**/95/152	-	-	0.56
linalyl acetate	40.25	1210	**43**/93/121	-	-	1.55
nerol	40.38	1227	**41**/69/154	35.85	-	-
carvone	40.747	1235	54/82/**93**/150	-	-	0.13
neral	41.31	1239	41/**69**/109/152	35.34	-	-
thymol	41.43	1281	**135**/150/65	-	-	0.66
bornyl acetate	41.91	1288	**41**/95/121	-	1.18	-
carvacrol	41.98	1298	41/**135**/150	-	-	0.51
eugenol	44.05	1356	43/131/149/**164**	-	3.03	-
neryl acetate	44.06	1359	41/**69**/93/154	7.54	-	-
methyl eugenol	45.03	1403	41/107/163/**178**	-	1.28	-
***Sesquiterpenes***						
α-bergamotene (E)(Z)	46.33	1432	41/**93**/119/204	-	3.2	-
β-caryophyllene (E)	46.48	1417	41/**93**/103/161	2.94	-	1.76
***Others***						
1-octen-3-ol	30.19	974	**43**/57	-	-	0.06
**Total area of all identified compounds (%)**				91.97	87	98.39
**Total Monoterpenes (%)**				89.03	83.8	96.57
**Total Sesquiterpenes (%)**				2.94	3.2	1.76
**Total Others (%)**				-	-	0.06

* Experimental Kovats index; ** in bold *m*/*z* used for quantification.

**Table 2:** Presence of compounds in fortified dairy products and transfers from milk to cheese and whey (*n* = 3).

	*Melissa officinalis*					*Ocimum basilicum*					*Thymus vulgaris*				
	Conc. (mg/kg) ^†^			Transf. (%) ^‡^		Conc. (mg/kg)			Transf. (%)		Conc. (mg/kg)			Transf. (%)	
	M§	C	W	C	W	M	C	W	C	W	M	C	W	C	W
Number of compounds	11	9	10			19	18	19			22	22	18		
*Monoterpene family*															
α-thujene	-	-	-	-	-	0.04	0.03	0.01	75.00	25.00	2.19	0.28	0.22	12.79	10.05
α-pinene	1.53	0.18	0.23	11.76	15.03	0.47	0.33	0.09	70.21	19.15	21.10	2.58	2.19	12.23	10.38
camphene	8.60	0.99	1.36	11.51	15.81	0.13	0.08	0.03	61.54	23.08	7.79	0.92	0.77	11.81	9.88
sabinene	-	-	-	-	-	0.45	0.21	0.08	46.67	17.78	5.64	0.64	0.64	11.35	11.35
β-pinene	0.22	0.03	0.05	13.64	22.73	0.67	0.32	0.14	47.76	20.90	34.08	3.72	4.25	10.31	11.78
α-phelandrene	-	-	-	-	-	-	-	-	-	-	3.26	0.40	0.40	12.27	12.27
α-terpinene	-	-	-	-	-	-	-	-	-	-	22.52	2.50	2.80	11.10	12.43
p-cymene	-	-	-	-	-	0.27	0.12	0.05	44.44	18.52	13.04	1.48	1.65	11.35	12.65
sylvestrene	-	-	-	-	-	-	-	-	-	-	16.54	1.56	-	9.43	-
1.8 cineole	-	-	-	-	-	15.49	4.55	4.37	29.37	28.21	5.73	0.61	2.70	10.65	47.12
β-ocimene (E)	0.48	0.06	0.09	12.50	18.75	1.28	0.47	0.26	36.72	20.31	0.45	0.06	-	13.33	-
γ-terpinene	-	-	-	-	-	0.10	0.04	0.03	40.00	30.00	27.88	3.10	3.95	11.12	14.17
4-thujanol (Z)	-	-	-	-	-	-	-	-	-	-	0.52	0.03	-	5.77	-
terpinolene	0.45	0.04	0.06	8.89	13.33	0.26	0.34	0.06	130.77	23.08	7.19	0.88	1.17	12.24	16.27
linalool	1.19	0.17	0.36	14.29	30.25	13.99	3.85	3.62	27.52	25.88	61.83	6.57	12.98	10.63	20.99
4-thujanol (E)	-	-	-	-	-	-	-	-	-	-	1.99	0.13	-	6.53	-
camphor	-	-	-	-	-	0.61	0.18	0.15	29.51	24.59	0.85	0.09	0.17	10.59	20.00
borneol	-	-	-	-	-	0.15	0.04	0.03	26.67	20.00	2.14	0.30	0.49	14.02	22.90
terpinen-4-ol	0.15	-	0.02	-	13.33	0.20	0.05	0.04	25.00	20.00	13.03	1.45	3.31	11.13	25.40
α-terpineol	-	-	-	-	-	-	-	-	-	-	1.26	0.18	0.40	14.29	31.75
4-allyl-anisole	-	-	-	-	-	47.02	12.70	9.97	27.01	21.20	-	-	-	-	-
linalyl acetate	-	-	-	-	-	-	-	-	-	-	0.61	0.08	-	13.11	-
nerol	17.56	0.86	3.57	4.90	20.33	-	-	-	-	-	-	-	-	-	-
neral	16.30	0.86	3.38	5.28	20.74	-	-	-	-	-	-	-	-	-	-
eugenol	-	-	-	-	-	0.77	-	0.07	-	9.09	-	-	-	-	-
bornyl acetate	-	-	-	-	-	0.64	0.18	0.12	28.13	18.75	0.17	0.02	0.08	11.76	47.06
neryl acetate	1.28	0.18	0.34	14.06	26.56	-	-	-	-	-	-	-	-	-	-
methyl eugenol	-	-	-	-	-	0.17	0.03	0.03	17.65	17.65	-	-	-	-	-
*Sesquiterpene family*															
α-bergamotene (E)(Z)	-	-	-	-	-	0.68	0.18	0.14	26.47	20.59	-	-	-	-	-
β-caryophyllene (E)	0.49	0.06	0.15	12.24	30.61	-	-	-	-	-	0.48	0.08	0.19	16.67	39.58
*Total identified compounds*	48.25	3.33	9.61	7.11	19.92	83.39	23.70	19.29	28.42	23.13	250.29	27.66	38.36	11.05	15.33
*Total monoterpenes*	47.76	3.37	9.46	7.06	19.81	82.71	23.52	19.15	28.44	23.15	249.81	27.58	38.17	11.04	15.28
*Total sesquiterpenes*	0.49	0.06	0.15	12.24	30.61	0.68	0.18	0.14	26.47	20.59	0.48	0.08	0.19	16.67	39.58

^†^ Concentration of the compound in the matrix expressed as mg/kg; ^‡^ Transfer of compounds from milk to cheese and whey; §M, C, W: Milk, Cheese, and Whey samples.
